# Supplementary figures and images for: Genome-Wide Analysis and Functional Characterization of LACS Gene Family Associated with Lipid Synthesis in Cotton (Gossypium spp.)
Source: Int J Mol Sci. 2023 May 10;24(10):8530. doi: 10.3390/ijms24108530 (PMC10218317; doi:10.3390/ijms24108530)

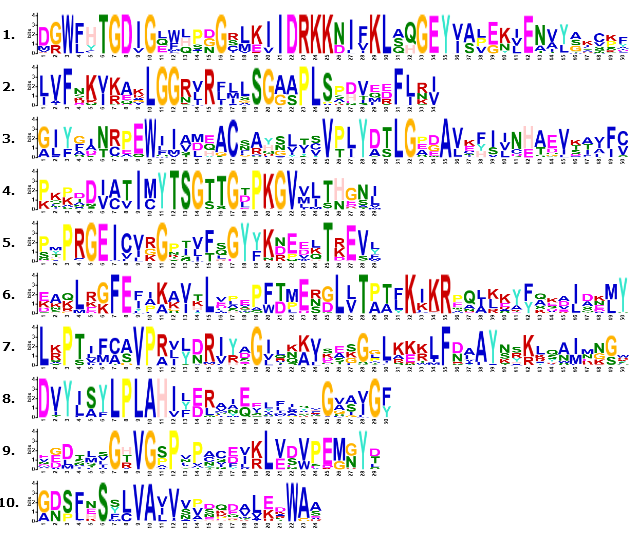

Supplement: Supplementary file 1 [file ijms-24-08530-s001.zip › Figure S1. The logos of 10 motifs in 65LACS proteins.tif]
